# Brown adipose tissue prevents glucose intolerance and cardiac remodeling in high-fat-fed mice after a mild myocardial infarction

**DOI:** 10.1038/s41366-021-00999-9

**Published:** 2021-10-29

**Authors:** Carmem Peres Valgas da Silva, Vikram K. Shettigar, Lisa A. Baer, Eaman Abay, Kendra L. Madaris, Mikayla R. Mehling, Diego Hernandez-Saavedra, Kelsey M. Pinckard, Nickolai P. Seculov, Mark T. Ziolo, Kristin I. Stanford

**Affiliations:** 1 Dorothy M. Davis Heart and Lung Research Institute, The Ohio State University Wexner Medical Center, Columbus, OH USA; 2 Department of Physiology and Cell Biology, The Ohio State University College of Medicine, Columbus, OH USA; 3 Department of Internal Medicine, The Ohio State University College of Medicine, Columbus, OH USA

**Keywords:** Cardiovascular diseases, Diabetes

## Abstract

**Background:**

Obesity increases the risk of developing impaired glucose tolerance (IGT) and type 2 diabetes (T2D) after myocardial infarction (MI). Brown adipose tissue (BAT) is important to combat obesity and T2D, and increasing BAT mass by transplantation improves glucose metabolism and cardiac function. The objective of this study was to determine if BAT had a protective effect on glucose tolerance and cardiac function in high-fat diet (HFD) fed mice subjected to a mild MI.

**Methods:**

Male C57BL/6 mice were fed a HFD for eight weeks and then divided into Sham (Sham-operated) and +BAT (mice receiving 0.1 g BAT into their visceral cavity). Sixteen weeks post-transplantation, mice were further subdivided into ±MI (Sham; Sham-MI; +BAT; +BAT-MI) and maintained on a HFD. Cardiac (echocardiography) and metabolic function (glucose and insulin tolerance tests, body composition and exercise tolerance) were assessed throughout 22 weeks post-MI. Quantitative PCR (qPCR) was performed to determine the expression of genes related to metabolic function of perigonadal adipose tissue (pgWAT), subcutaneous white adipose tissue (scWAT), liver, heart, tibialis anterior skeletal muscle (TA); and BAT.

**Results:**

+BAT prevented the increase in left ventricle mass (LVM) and exercise intolerance in response to MI. Similar to what is observed in humans, Sham-MI mice developed IGT post-MI, but this was negated in +BAT-MI mice. IGT was independent of changes in body composition. Genes involved in inflammation, insulin resistance, and metabolism were significantly altered in pgWAT, scWAT, and liver in Sham-MI mice compared to all other groups.

**Conclusions:**

BAT transplantation prevents IGT, the increase in LVM, and exercise intolerance following MI. MI alters the expression of several metabolic-related genes in WAT and liver in Sham-MI mice, suggesting that these tissues may contribute to the impaired metabolic response. Increasing BAT may be an important intervention to prevent the development of IGT or T2D and cardiac remodeling in obese patients post-MI.

## Introduction

Obesity is a complex disease that is rapidly increasing in the United States and worldwide [1]. Obesity affects whole-body metabolism and is associated with an increased risk of type 2 diabetes (T2D) and cardiovascular disease (CVD), including myocardial infarction (MI) [2]. MI occurs when reduced blood flow to the heart causes myocardial injury due to lack of oxygen [3]. The ischemic heart undergoes loss of cardiomyocytes, left ventricular (LV) remodeling, and hypertrophy, all of which play an important role in the progression of heart failure (HF) [4]. Although advances in cardiovascular research have resulted in significantly reduced mortality rates in MI patients over the last few decades [5], patients who survive an MI continue to require appropriate interventions to prevent substantial disability and recurrent coronary events [5, 6].

There are several risk factors that determine the severity of MI, including advanced age, coexistence of previous coronary disease, and metabolic disease, including obesity and type 2 diabetes [7–9]. Several recent studies have demonstrated that obese patients are more likely to develop insulin resistance (IR) and impaired glucose tolerance (IGT) post-MI [10–14], but the reasons for this are not completely understood. It has been postulated that MI-induced catecholamine stress alters glucose and lipid metabolism, resulting in impaired insulin sensitivity in peripheral tissues such as white adipose tissue, liver, and skeletal muscle [13]. Patients with visceral obesity are more likely to develop IGT and type 2 diabetes, which in turn increases the likelihood of recurrent cardiac ischemic events post-MI [13–15]. In fact, IR, IGT, and dysglycemia may also contribute to the development of adverse LV remodeling post-MI [14, 16] which is recognized as the predominant pathological process in the development of heart failure [17]. Furthermore, studies have shown direct association between IR, IGT, type 2 diabetes, and increased risk of subsequent MIs and the development of heart failure post-MI [11, 12, 18]. Thus, new therapeutic strategies to reduce the risk of MI-induced insulin resistance, glucose intolerance, and type 2 diabetes are essential to improve life expectancy and quality of life in patients who suffer from MI.

An important tissue to combat the development of obesity, glucose intolerance, and insulin resistance is brown adipose tissue (BAT) [19, 20]. Upon activation, BAT increases energy expenditure and promotes glucose and fatty acid uptake, making the tissue a potential therapeutic target for patients with obesity, IR, and type 2 diabetes [20]. While BAT mass and activity are reduced with obesity [21, 22], studies have shown that increasing BAT mass by transplantation improves glucose metabolism and insulin sensitivity in rodents [23, 24]. Recent work from our laboratory identified a role for BAT to mediate cardiac function [25], but the effects of BAT on metabolic health or cardiac function after MI have not been investigated.

Here, we investigated a role for BAT transplantation (+BAT) to preserve cardiac health and protect against the development of glucose intolerance post-MI in mice. Our results show that while there was no difference in cardiac ejection fraction, MI increased LV mass (LVM) and reduced exercise tolerance at 22 weeks post-MI. The LV hypertrophy and exercise intolerance were not seen in MI mice who received BAT transplantation (+BAT-MI). Moreover, MI mice who did not receive BAT (Sham-MI) developed glucose intolerance at 22 weeks post-MI, while +BAT-MI mice were completely protected from the development of glucose intolerance. MI increased the expression of several genes related to inflammation, lipid, and glucose metabolism in the perigonadal white adipose tissue (pgWAT) and liver of Sham-MI mice, but BAT transplantation (+BAT-MI) negated these alterations. These data indicate that increasing BAT protects against the detrimental effects of MI on LV hypertrophy, exercise intolerance, and impaired glucose metabolism, providing a potential therapeutic role for BAT with significant translational ramifications.

## Experimental methods

### Mice and BAT transplantation

**Fig. 1:**
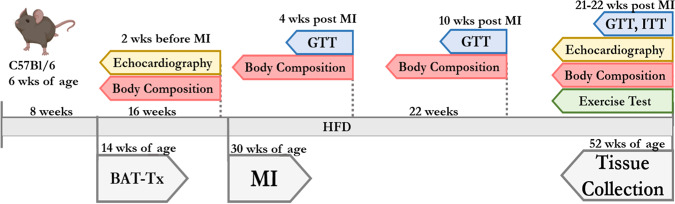
Experimental design. Six-week-old C57BL/6 mice were placed on a HFD for 8 weeks then subjected to brown adipose tissue transplantation (BAT-Tx). Sixteen-weeks post BAT-Tx transplantation mice were subjected to either a Sham or myocardial infarction (MI) surgery. Two weeks before MI, mice were subjected to echocardiography and body composition assessment. At 4, 10, and 22 post MI, mice were subjected to body composition assessment, and glucose tolerance tests (GTT). Insulin tolerance tests (ITT) and exercise tolerance tests were performed at 21 weeks post MI, and echocardiography was performed at 22 weeks post MI. At 22 weeks post MI, mice were euthanized, and tissues were collected for assessment of gene expression and biochemical methods.

Male C57Bl/6 mice (six- weeks old) (Charles River Laboratories) were placed on a high-fat diet (HFD) (60% kcal from fat) for eight weeks and then divided into two groups: Sham operated (Sham) or mice that received BAT transplantation (+BAT). Transplantation of BAT was performed as previously described [23, 25]. Briefly, aged-matched male C57BL/6 donor mice were euthanized with isoflurane followed by cervical dislocation, BAT was removed from the interscapular region and incubated in a solution of 10 mL saline at 37 °C for 20–30 min. Recipient mice (+BAT) were anesthetized by isoflurane inhalation in oxygen (3% isoflurane in 97% oxygen). For each recipient mouse, 0.1 g donor BAT was transplanted into the visceral cavity. The transplant was carefully lodged deep between folds within the endogenous epididymal fat of the recipient. Mice that were sham-operated (Sham) underwent the same procedure, but instead of receiving BAT, their epididymal fat pad was located, exposed, and then replaced. Mice were maintained on an HFD throughout 52 weeks of age (22 weeks post-MI). The experimental design is shown in Fig. 1.

### Myocardial Infarction

Sixteen weeks post-transplantation, mice were further subdivided into four groups with half of the mice undergoing myocardial infarction (MI) surgery: Sham, +BAT, Sham-MI, and +BAT-MI. MI was induced in the Sham-MI and +BAT-MI mice by a low suture in the left anterior descending (LAD) coronary artery, as described previously [26, 27]. Briefly, permanent MI surgery was performed by anaesthetizing mice with 2% isoflurane in 98% O_2_ and mechanical ventilation. After a left thoracotomy, the fourth intercostal space and the lungs were retracted. The LAD coronary artery was permanently ligated with an 8-0 silk low suture 0.6 mm distal to the atrioventricular junction [27]. The low occlusion allowed for a substantial proportion of the left ventricle to remain perfused and functional, representing a mild MI [27]. The Sham and +BAT groups were subjected to all the procedures except the LAD ligation. Post-MI echocardiography was performed at 22 weeks post MI, and all mice determined to be in heart failure (ejection fraction of <35) post-MI were removed from the study and did not undergo further analyses.

### In vivo cardiac function

All mice underwent echocardiography at baseline and 22 weeks post MI. Mice were anesthetized with 1-2% isoflurane and echocardiography was conducted using a Vevo 2100 Ultrasound as described previously [26]. Echocardiogram data was analyzed using VevoLab software to determine left ventricle (LV) ejection fraction, LV mass, End Diastolic Dimension (EDD), End Diastolic Volume (EDV), and LV diastolic diameter.

### Exercise tolerance test

Exercise tolerance for cardiovascular fitness was determined by an exercise tolerance test [28]. At 20 weeks post-MI, all mice were first acclimated to the treadmill for three consecutive days. During acclimation, mice were placed in the treadmill for 3 min, after which the shock grid was activated (3 Hz and 1.5 mA). Next, the treadmill was engaged to a walking speed of 6 m/min for 5 min and progressively increased up to 12 m/min for a total duration of 12 min of exercise. After one week of acclimation, mice were subjected to the exercise tolerance test, which consisted of placing the mice on the treadmill at 0° incline with the shock grid activated. The treadmill speeds were then increased until exhaustion as follows: (speed, duration, grade)—(0 m/min, 3 min, 0°), (6 m/min, 3 min, 0°), (9 m/min, 3 min, 5°), (12 m/min, 3 min, 10°), (15 m/min, 3 min, 15°), (18, 21, 23, 24 m/min, 3 min, 15°), and (+1 m/min, each 1 min thereafter, 15°). The endpoint for treadmill cessation was defined as the time at which mice maintained continuous contact with the shock grid for 5 s [28].

### Metabolic characterization

Body weight was measured weekly. Food intake was measured over the first 10 weeks post MI. Body composition was determined using EchoMRI (EchoMRI^TM^3-in-1) at two weeks before MI, and 4, 10, and 22 weeks post MI. Glucose tolerance tests (GTT) were performed at 4, 10, and 22 weeks post MI. Mice fasted for 11 h (22:00–9:00) with free access to drinking water [23]. A baseline blood sample was collected from the tail of fully conscious mice, followed by i.p. injection of glucose (2.0 g/kg body weight), and blood was taken from the tail at 15, 30, 60, and 120 min after injection. Insulin tolerance tests (ITTs) were performed 21 weeks post-MI. Mice were fasted for 2 h (10:00–12:00), and baseline blood samples were collected from the tail of fully conscious mice. Insulin (1 U/kg body weight) (Humulin; Eli Lilly) was administered by i.p. injection, and blood samples were taken from the tail at 10, 15, 30, 45, and 60 min after injection [23]. Glucose concentrations were determined using an OneTouch Ultra-portable glucometer (LifeScan).

### Biochemical methods

Tissue processing and quantitative PCR (qPCR) were performed on tissue isolated from mice that were sacrificed at 52 weeks of age (22 weeks post-MI) as previously described [29]. The tissue was flash frozen and stored at −80 °C until processing. mRNA was measured by qRT-PCR (Roche LightCycler 480II) using SYBR Green detection (QuantaBio). Sigma-Aldrich custom primers were used for genes of interest with the sequences shown in Supplementary Table 1 [30]. Gene expression was normalized to the housekeeping gene GAPDH (perigonadal WAT, subcutaneous WAT, tibialis anterior skeletal muscle, and BAT), TBP (Liver), or RPL7a (heart). Circulating plasma insulin, triglycerides, and total cholesterol were measured using standard ELISA kits (Cayman Chemicals).

### Statistical analysis

The data are presented as means ± SEM. One-way and Two-way ANOVA with Tukey post hoc analysis were performed using GraphPad Prism software 7.0 (GraphPad Prism Software Inc., San Diego-CA). Student *t*-tests were performed to compare differences between Sham vs +BAT mice at 12 weeks post-transplantation (prior to MI). Kaplan–Meyer survival curve was plotted to identify the survival rate of Sham-MI vs +BAT-MI. Values of *P* < 0.05 were considered statistically significant.

## Results

### BAT transplantation minimally affected cardiac function post-MI

**Fig. 2:**
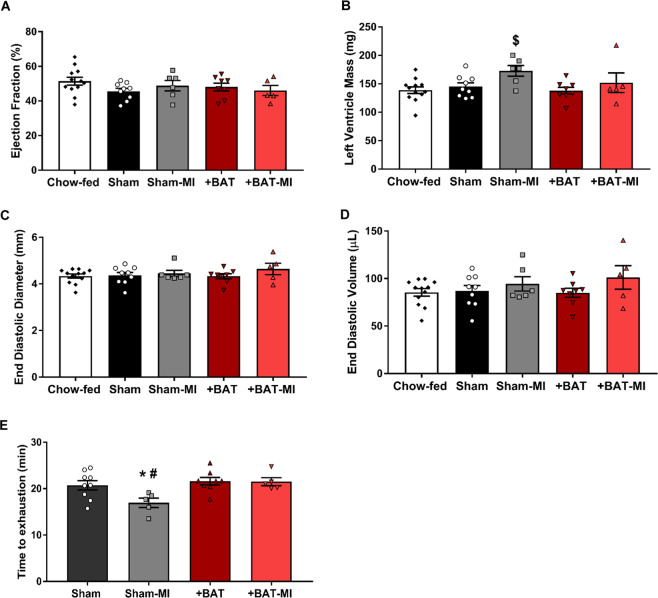
BAT transplantation minimally affected cardiac function post-MI. Cardiac function and structure were measured by (**A**) ejection fraction, (**B**) left ventricle mass, (**C**) end diastolic diameter, (**D**) end diastolic volume, and (**E**) time to exhaustion in exercise test in Chow-fed (*n* = 12), Sham (*n* = 9), Sham-MI (*n* = 6), +BAT (*n* = 10) and +BAT-MI (*n* = 5) mice that had EF over 35%. Data are presented as mean + S.E.M. One-way ANOVA was used with Tukey’s multiple comparisons tests. * represents *p* < 0.05 compared to Sham mice; # represents *p* < 0.05 compared to BAT-MI mice, and $ represents *p* < 0.05 compared to Chow-fed mice.

To determine the effect of BAT transplantation on the cardiac function post-MI, echocardiography was performed in aged-matched Chow-fed, Sham, Sham-MI, +BAT, and +BAT-MI mice at baseline and 22 weeks post MI. There was no difference in ejection fraction measurements prior to MI between Chow-fed, Sham, and +BAT groups, but multiple parameters of cardiac remodeling were adversely affected by a high-fat diet (Figure S1A; Supplemental Table 2). We hypothesized that a severe MI would have a detrimental effect on cardiac function that could not be rescued by BAT transplantation, thus we were interested in investigating the effects of mild-MI. Because of this, we excluded mice who were in heart failure, or those with an ejection fraction (EF) of less than 35% (Figure S1B). This included Sham mice with an EF < 35, which was likely due to a chronic high-fat diet over a 46 week period. There were no +BAT mice with an EF less than 35%. There was a difference in heart failure incidence among MI groups, with more +BAT-MI mice having an EF < 35 (Figure S1B), however, this is likely because more Sham-MI mice died in the first week post surgery and throughout the study compared to +BAT-MI mice (Figure S1C). There was no difference in EF among groups that had an EF > 35%, including no effect of HFD or increasing BAT (+BAT) when compared to age-matched chow-fed controls (Fig. 2A).

MI leads to adverse cardiac remodeling and impaired cardiac function over time [31]. To determine if cardiac remodeling was affected by BAT transplantation or HFD, left ventricular mass (LVM), end diastolic diameter (EDD), end diastolic volume (EDV), and other cardiac parameters were measured 22 weeks post-MI (Fig. 2B–D; Supplemental Table 3). LVM was increased in Sham-MI mice compared to chow-fed, aged-matched mice, but this was not seen in +BAT-MI mice (Fig. 2B–D; Supplemental Table 3). There was no additional effect of MI or BAT transplantation on EDD or EDV. These data indicate a protective role of BAT transplantation on LV hypertrophy post-MI.

### BAT preserves exercise tolerance post-MI

MI results in exercise intolerance in humans and rodents [32, 33]. To determine if BAT protects against the MI-induced exercise intolerance in mice, mice underwent exercise tolerance test at 21-weeks post MI [28]. Exercise tolerance was decreased in Sham-MI mice compared to all other groups. Of importance, the +BAT-MI mice were protected from this effect, as they had a similar exercise tolerance to Sham and +BAT mice (Fig. 2E). Exercise intolerance is a hallmark of the development of heart failure post-MI [34, 35], and these data indicate that BAT preserves exercise tolerance at 21 weeks post-MI.

### BAT protects against glucose intolerance induced by MI

**Fig. 3:**
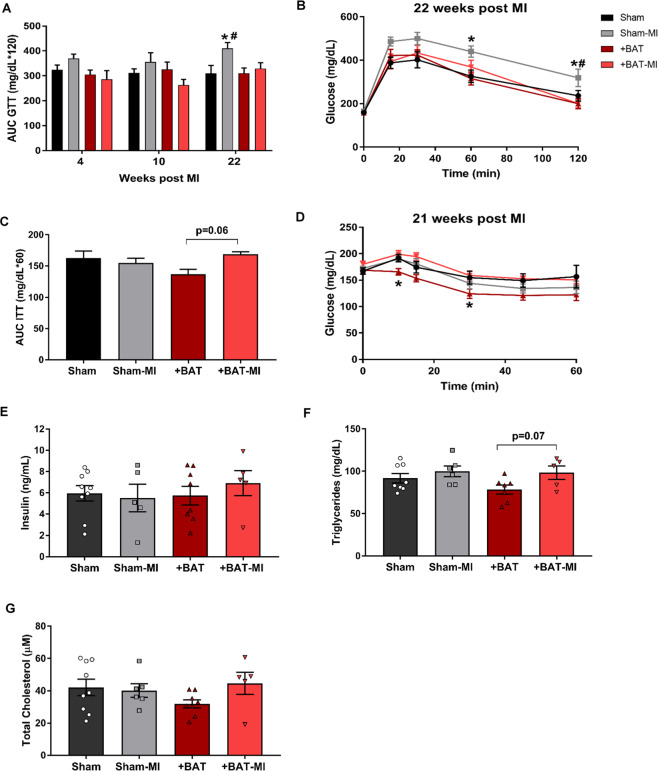
BAT transplantation protects against glucose intolerance induced by MI. Whole-body glucose homeostasis was assessed by glucose tolerance tests (GTT), and insulin tolerance test (ITT). **A** Area under curve (AUC) of glucose tolerance test (GTT) at weeks 4, 10, and 22 weeks post MI. **B** GTT excursion curve at 22 weeks post MI. **C** Insulin tolerance test (ITT) AUC at 21-weeks post MI. **D** ITT excursion curve at 21 weeks post MI. Biochemical measurements were performed after euthanasia at 22 weeks post MI: (**E**) Fasting insulin, (**F**) fasting triglycerides, and (**G**) total cholesterol (Sham *n* = 9, Sham-MI *n* = 5, +BAT *n* = 8, +BAT-MI *n* = 5). Data are presented as mean + S.E.M. Two-way ANOVA was used with Tukey’s multiple comparisons tests for (**A**, **C**, **E**, **F**, and **G**). Repeated measures two-way ANOVA was used for (**B**) and (**D**) with Tukey’s multiple comparisons tests. * symbols represent difference of Sham-MI or +BAT vs. Sham mice (*p < 0.05). # symbols represent difference of Sham-MI versus +BAT-MI mice (#*p* < 0.05).

Obese humans develop insulin resistance (IR) and impaired glucose tolerance (IGT) one year after a mild MI [13]. The onset of IR is associated with activated inflammatory response and increased circulating fatty acids due to catecholamine stress caused by MI injury [11, 13]. BAT mediates whole-body glucose and insulin sensitivity, and increasing BAT by transplantation improves whole-body glucose homeostasis in mice [23]. Thus, we investigated whether BAT protects against the MI-induced impairment in glucose tolerance. Prior to MI, there was no difference in glucose tolerance between Sham and +BAT mice, but insulin tolerance was improved in +BAT mice (Figure 2SA-B). Similar to what is observed in humans [13], Sham-MI mice developed a progressive worsening of glucose tolerance compared to all other groups (Fig. 3A), and it was most prominent at 22 weeks post-MI (Fig. 3A, B). This impairment in glucose tolerance was completely negated by BAT transplantation in +BAT-MI mice (Fig. 3A, B). In fact, glucose tolerance in +BAT-MI mice was similar to Sham and +BAT mice. This preservation in glucose tolerance was independent of changes in body weight, fat mass, lean mass, and food intake, which were not different among groups (Figure S2C–F).

To determine if MI affected insulin sensitivity in mice, insulin tolerance was measured 21 weeks post-MI. +BAT mice had improved insulin tolerance compared to Sham at 22 weeks post-MI, but there was no effect of MI on insulin tolerance (Fig. 3C, D). Biochemical markers such as serum insulin, triglycerides, and total cholesterol were measured to determine if they were altered by MI or BAT transplantation. There was no effect of MI or BAT transplantation on circulating insulin, triglycerides or total cholesterol 22 weeks post MI (Fig. 3E–G).

### BAT transplantation negates the effect of MI on the expression of genes involved in inflammation and insulin resistance in white adipose tissue and liver

**Fig. 4:**
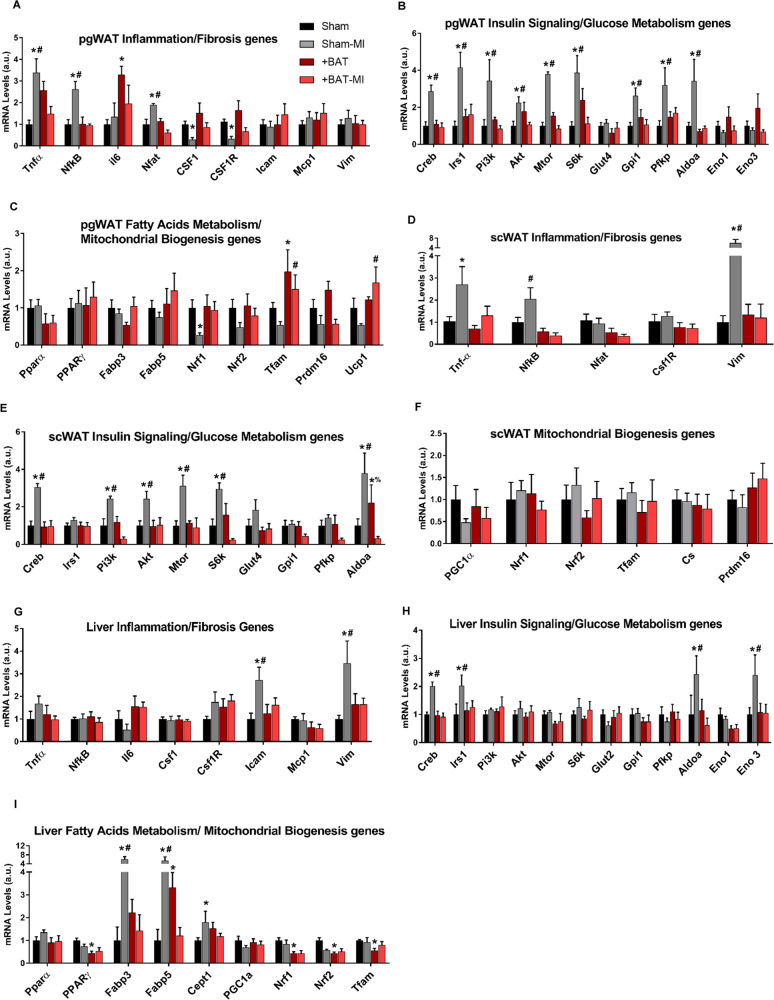
BAT attenuates expression of genes involved in inflammation and insulin resistance increased by MI in white adipose tissue and liver. Quantitative PCR (qPCR) was performed on tissue isolated from mice that were sacrificed at 52 weeks of age (22 weeks post-MI). Genes related to inflammation and fibrosis, insulin signaling and glucose metabolism, fatty acid metabolism, and mitochondrial biogenesis were accessed in (**A**–**C**) pgWAT, (**D**–**F**) scWAT, and (**G**–**I**) liver of Sham (*n* = 8), Sham-MI (*n* = 3–5), +BAT (*n* = 5–8), and +BAT-MI (*n* = 4–5). Data are presented as mean + S.E.M (*n* = 5–9/group). Two-way ANOVA was used with Tukey’s multiple comparisons tests. *symbols represent the difference of Sham-MI or +BAT vs. Sham mice (*p < 0.05). # symbols represent difference of Sham-MI versus +BAT-MI mice (#*p* < 0.05).

Given the protective role of BAT transplantation on glucose tolerance post-MI, we investigated possible mechanisms and tissues that mediate the improved whole-body glucose tolerance. We analyzed genes involved in inflammation, fibrosis, redox state, senescence, insulin signaling, glucose and lipid metabolism, mitochondrial biogenesis and mitochondrial function in perigonadal white adipose tissue (pgWAT), subcutaneous white adipose tissue (scWAT), liver, brown adipose tissue (BAT), tibialis anterior skeletal muscle (TA) and heart. In total, expression of 93 genes were measured (Supplemental Table 1). There were striking increases in several genes involved in inflammation (*Tnf-α; NfkB; Nfat*), insulin resistance (*Creb*), insulin signaling (*Irs1; Pi3k; Akt; Mtor; S6k*); and glucose metabolism (*Gpi1; Pfkp; Aldoa*) in response to MI in pgWAT and scWAT (Fig. 4A–F, Supplemental Fig. 3A–F). There were also multiple genes involved in mitochondrial biogenesis (*Nrf1;Nrf2*) and macrophage differentiation (*Csf1*; *Csf1R*) that were decreased in pgWAT in response to MI. Expression of these genes was not altered in +BAT-MI mice (Fig. 4A–F, Supplemental Fig. 3A–F).

In the liver, expression of select genes related to fibrosis (*Icam; Vim*); gluconeogenesis (*Creb*); glucose metabolism (*Aldoa*; *Eno3*), and lipid transport (*Fabp3; Fabp5*) were increased in Sham-MI mice compared to all other groups (Fig. 4G–I; Supplemental Figs. 3G–J, 4A,B). Importantly, BAT transplantation in MI mice negated this effect in the liver (Fig. 4G–I). In the heart, markers of fibrosis including *Mmp9*, *S6k,* and *Ddr2* were increased in Sham-MI mice, and this effect was suppressed by BAT transplantation in +BAT-MI mice (Supplemental Fig. 4D). The expression of the antioxidant enzymes *SOD1-3* was increased in +BAT-MI compared to all groups (Supplemental Fig. 4C–G). Minimal effects of MI or BAT transplantation were observed in TA (Supplemental figure 5A–D) and interscapular BAT (Supplemental Figures 5E–H). Together these data suggest that mild MI alters gene expression in peripheral tissues including pgWAT, scWAT and liver, which could contribute to the impaired whole-body glucose metabolism. BAT transplantation protects against these adverse effects, which might be mediated through improvements in peripheral tissue inflammation, insulin signaling, and mitochondrial metabolism.

## Discussion

Here, we investigated the protective role of BAT against the detrimental effects of MI on LV hypertrophy and glucose metabolism in male mice. We found that increasing BAT mass by transplantation prevents the increased LV mass, decreased exercise tolerance, and impaired glucose tolerance after a mild MI in a mouse model of HFD-induced obesity.

Obesity is caused by alterations in energy balance in which energy intake exceeds energy expenditure [36]. This imbalance leads to adipose tissue accumulation and metabolic alterations that can result in cardiovascular disease (CVD) and type 2 diabetes [1, 37, 38]. Studies in rodents and humans have demonstrated that increased BAT mass is associated with improved metabolic health [19, 20, 39], and BAT mass and activity are decreased with obesity [21, 22]. A recent study in human subjects has shown that reduced BAT mass is associated with increased incidence of T2D and cardiovascular disease [40]. We and others have previously shown that increasing BAT mass by transplantation improves metabolic health in obese rodents [23, 24, 41–43]. Thus, the objective of this study was to determine if obese mice with increased BAT mass are protected against progressive glucose intolerance and cardiac hypertrophy after a MI. In the current study, we demonstrated that the long-term beneficial effects of BAT transplantation on glucose metabolism are independent of body mass and composition. Thus, increasing BAT can prevent long-term metabolic alterations which could have beneficial implications for obese patients with CVD.

The number of patients that survive after an MI has increased over the last few decades [5, 44]. The patients who survive a mild MI need preventive care for heart failure and treatment for the long-term metabolic alterations, including development of IR and IGT, caused by MI [5, 7, 8, 10–15]. In fact, abnormal glucose tolerance is an independent predictor of cardiovascular events such as recurrent MI, stroke, adverse cardiac remodeling, and heart failure during a median follow-up time up to 34 months post MI [18, 45–47]. Similar to what is observed in humans [13], we show that mild MI led to progressive glucose intolerance in obese mice. Our study demonstrated that increasing BAT mass prevents the development of IGT in response to MI. These data represent a potential therapeutic approach, protecting against the development of IGT and, if applicable to humans, potentially preventing recurring cardiovascular events and development of heart failure that arise following IGT and type 2 diabetes.

Exercise intolerance is a hallmark of heart failure and is associated with a poor quality of life and increased mortality [48]. Exercise intolerance is one of the primary chronic symptoms in patients with heart failure with preserved ejection fraction, and is associated with poor prognosis post MI [34, 35, 49, 50]. It is well known that LV remodeling, including LV hypertrophy, may contribute to exercise intolerance post MI [51, 52]. In our study, BAT transplantation prevented the increased LV mass and exercise intolerance caused by mild MI. Taken together, these data suggest a potential role for BAT to prevent poor cardiac outcomes after a mild myocardial infarction.

The mechanisms for IR and IGT to develop as a result of MI have not been fully established. It has been suggested that the post-MI catecholamine stress induces hyperglycemic, inflammatory and lipolytic response, progressively affecting metabolism in peripheral tissues, and may lead to the development of IR, IGT, and type 2 diabetes, especially in obese subjects [11, 13, 53–55]. BAT activation consumes substantial amounts of glucose as fuel for thermogenesis, contributing significantly to whole-body glucose disposal [56]. There were no differences in the pre-MI GTT, or in basal glucose among groups at 22 weeks post-MI. This, as well as previous studies from our laboratory [23] indicate that the transplanted BAT is not acting just as a glucose ‘sink’. These data indicate that there is a complex endocrine/paracrine signaling network where increasing BAT mass affects other tissues and improves systemic glucose metabolism. To investigate potential mechanisms for BAT to protect against the development of IGT post-MI, we assessed the expression of more than 90 genes in multiple tissues. Several genes were altered by MI in WAT and liver, but were not changed in +BAT-MI mice. In both pgWAT and scWAT, MI increased expression of the inflammatory markers *Tnf-α* and *NfkB*. These inflammatory markers are associated with increased local and systemic IR and the development of type 2 diabetes [57, 58]. Several other genes associated with the onset of IR were altered in WAT of Sham-MI mice, such as the macrophage differentiation genes *Csf1* and *Csf1R* [54], and the pathway *Pi3k/Akt/Mtor/S6k* [59–62]. The cAMP-responsive transcription factor CREB is associated with insulin resistance and gluconeogenesis [63–65], and it was upregulated in both WAT and liver of Sham-MI mice but not in +BAT-MI mice. Interestingly, in the absence of MI, BAT transplantation had minimal effect in the adipose tissue, and did not affect liver gene expression, suggesting that BAT transplantation affects the metabolic phenotype that is specifically induced by MI. These data indicate that multiple pathways in WAT and liver could be involved in the development of IGT post-MI and that increasing BAT prior to MI affected tissue crosstalk by preventing MI-induced alterations in WAT and liver. Further studies will investigate mechanistically the effects of MI on the glucose metabolism and the protective role of BAT.

This is the first study to demonstrate that increasing BAT mass by transplantation prevents impaired glucose tolerance in HFD-fed mice and is cardioprotective after a mild MI in male mice. This is likely associated with the upregulation of several metabolic genes induced by mild MI in WAT and liver gene regulation in the heart and is independent of body composition. The mechanism through which BAT exerts this protective effect on increased LV mass, impaired glucose tolerance, insulin resistance, and type 2 diabetes is not clear and will be the focus of future investigations. Together these data identify a therapeutic role for BAT to mediate glucose metabolism post-MI and indicate that increasing BAT could have significant translatable potential and great impact on public health.

## Supplementary information

Supplemental Figure Legends

Supplemental Tables

Supplemental Figure 1

Supplemental Figure 2

Supplemental Figure 3

Supplemental Figure 4

Supplemental Figure 5

## References

[1] Lavie CJ, De Schutter A, Parto P, Jahangir E, Kokkinos P, Ortega FB (2016). Obesity and prevalence of cardiovascular diseases and prognosis-the obesity paradox updated. Prog Cardiovasc Dis.

[2] Pinckard K, Baskin KK, Stanford KI (2019). Effects of exercise to improve cardiovascular health. Front Cardiovasc Med.

[3] Lu L, Liu M, Sun R, Zheng Y, Zhang P (2015). Myocardial infarction: symptoms and treatments. Cell Biochem Biophys.

[4] Cahill TJ, Kharbanda RK (2017). Heart failure after myocardial infarction in the era of primary percutaneous coronary intervention: mechanisms, incidence and identification of patients at risk. World J Cardiol.

[5] Frampton J, Devries JT, Welch TD, Gersh BJ (2020). Modern management of ST-segment elevation myocardial infarction. Curr Probl Cardiol.

[6] Thygesen K, Alpert JS, White HD (2007). Universal definition of myocardial infarction. J Am Coll Cardiol.

[7] Myftiu S, Bara P, Sharka I, Shkoza A, Belshi X, Rruci E (2016). Heart failure predictors in a group of patients with myocardial infarction. Open Access Maced J Med Sci.

[8] Spencer FA, Meyer TE, Gore JM, Goldberg RJ (2002). Heterogeneity in the management and outcomes of patients with acute myocardial infarction complicated by heart failure: the National Registry of Myocardial Infarction. Circulation.

[9] Shinlapawittayatorn K, Chattipakorn SC, Chattipakorn N (2018). The influence of obese insulin-resistance on the outcome of the ischemia/reperfusion insult to the heart. Curr Med Chem.

[10] Nishio K, Shigemitsu M, Kusuyama T, Fukui T, Kawamura K, Itoh S (2006). Insulin resistance in nondiabetic patients with acute myocardial infarction. Cardiovasc Revasc Med.

[11] Gruzdeva O, Uchasova E, Dyleva Y, Belik E, Shurygina E, Barbarash O (2013). Insulin resistance and inflammation markers in myocardial infarction. J Inflamm Res.

[12] Fu F, Zhao K, Li J, Xu J, Zhang Y, Liu C (2015). Direct evidence that myocardial insulin resistance following myocardial ischemia contributes to post-ischemic heart failure. Sci Rep.

[13] Barbarash O, Gruzdeva O, Uchasova E, Dyleva Y, Belik E, Akbasheva O (2016). The role of adipose tissue and adipokines in the manifestation of type 2 diabetes in the long-term period following myocardial infarction. Diabetol Metab Syndr.

[14] Yang CD, Shen Y, Lu L, Ding FH, Yang ZK, Zhang RY (2019). Insulin resistance and dysglycemia are associated with left ventricular remodeling after myocardial infarction in non-diabetic patients. Cardiovasc Diabetol.

[15] Terlecki M, Bryniarski L, Bednarek A, Kocowska M, Kawecka-Jaszcz K, Czarnecka D (2015). The risk of diabetes development in long-term observation of patients with acute hyperglycaemia during myocardial infarction. Kardiol Pol.

[16] Sundström J, Lind L, Nyström N, Zethelius B, Andrén B, Hales CN (2000). Left ventricular concentric remodeling rather than left ventricular hypertrophy is related to the insulin resistance syndrome in elderly men. Circulation.

[17] Yang CD, Shen Y, Ding FH, Yang ZK, Hu J, Shen WF (2020). Visit-to-visit fasting plasma glucose variability is associated with left ventricular adverse remodeling in diabetic patients with STEMI. Cardiovasc Diabetol.

[18] Szepietowska B, McNitt S, Kutyifa V, Ryan D, Corsetti J, Sparks C (2015). Insulin resistance predicts the risk for recurrent coronary events in post-infarction patients. Cardiol J.

[19] Nedergaard J, Bengtsson T, Cannon B (2007). Unexpected evidence for active brown adipose tissue in adult humans. Am J Physiol Endocrinol Metab.

[20] Peres Valgas da Silva C, Hernández-Saavedra D, White JD, Stanford KI (2019). Cold and exercise: therapeutic tools to activate brown adipose tissue and combat obesity. Biology (Basel).

[21] Vijgen GH, Bouvy ND, Teule GJ, Brans B, Schrauwen P, van Marken Lichtenbelt WD (2011). Brown adipose tissue in morbidly obese subjects. PLoS One.

[22] van Marken Lichtenbelt W (2011). Human brown fat and obesity: methodological aspects. Front Endocrinol (Lausanne).

[23] Stanford KI, Middelbeek RJ, Townsend KL, An D, Nygaard EB, Hitchcox KM (2013). Brown adipose tissue regulates glucose homeostasis and insulin sensitivity. J Clin Invest.

[24] White JD, Dewal RS, Stanford KI (2019). The beneficial effects of brown adipose tissue transplantation. Mol Aspects Med.

[25] Pinckard KM, Shettigar VK, Wright KR, Abay E, Baer LA, Vidal P (2020). A novel endocrine role the BAT-released lipokine 12,13-diHOME to mediate cardiac function. circulation.

[26] Shettigar V, Zhang B, Little SC, Salhi HE, Hansen BJ, Li N (2016). Rationally engineered Troponin C modulates in vivo cardiac function and performance in health and disease. Nat Commun.

[27] Degabriele NM, Griesenbach U, Sato K, Post MJ, Zhu J, Williams J (2004). Critical appraisal of the mouse model of myocardial infarction. Exp Physiol.

[28] Petrosino JM, Heiss VJ, Maurya SK, Kalyanasundaram A, Periasamy M, LaFountain RA (2016). Graded maximal exercise testing to assess mouse cardio-metabolic phenotypes. PLoS One.

[29] Lessard SJ, Rivas DA, Alves-Wagner AB, Hirshman MF, Gallagher IJ, Constantin-Teodosiu D (2013). Resistance to aerobic exercise training causes metabolic dysfunction and reveals novel exercise-regulated signaling networks. Diabetes.

[30] Stanford KI, Takahashi H, So K, Alves-Wagner AB, Prince NB, Lehnig AC (2017). Maternal exercise improves glucose tolerance in female offspring. Diabetes.

[31] Bhatt AS, Ambrosy AP, Velazquez EJ (2017). Adverse remodeling and reverse remodeling after myocardial infarction. Curr Cardiol Rep.

[32] Sumide T, Shimada K, Ohmura H, Onishi T, Kawakami K, Masaki Y (2009). Relationship between exercise tolerance and muscle strength following cardiac rehabilitation: comparison of patients after cardiac surgery and patients with myocardial infarction. J Cardiol.

[33] Pinsky JL, Jette AM, Branch LG, Kannel WB, Feinleib M (1990). The Framingham Disability Study: relationship of various coronary heart disease manifestations to disability in older persons living in the community. Am J Public Health.

[34] Fontes-Carvalho R, Sampaio F, Teixeira M, Rocha-Gonçalves F, Gama V, Azevedo A (2015). Left ventricular diastolic dysfunction and E/E’ ratio as the strongest echocardiographic predictors of reduced exercise capacity after acute myocardial infarction. Clin Cardiol.

[35] Lamas GA, Vaughan DE, Parisi AF, Pfeffer MA (1989). Effects of left ventricular shape and captopril therapy on exercise capacity after anterior wall acute myocardial infarction. Am J Cardiol.

[36] Hill JO, Wyatt HR, Peters JC (2012). Energy balance and obesity. Circulation.

[37] Kelley DE, Mokan M, Mandarino LJ (1992). Intracellular defects in glucose metabolism in obese patients with NIDDM. Diabetes.

[38] Goodpaster BH, Sparks LM (2017). Metabolic flexibility in health and disease. Cell Metab.

[39] Bouillaud F, Ricquier D, Thibault J, Weissenbach J (1985). Molecular approach to thermogenesis in brown adipose tissue: cDNA cloning of the mitochondrial uncoupling protein. Proc Natl Acad Sci USA.

[40] Becher T, Palanisamy S, Kramer DJ, Eljalby M, Marx SJ, Wibmer AG (2021). Brown adipose tissue is associated with cardiometabolic health. Nat Med.

[41] Liu X, Wang S, You Y, Meng M, Zheng Z, Dong M (2015). Brown adipose tissue transplantation reverses obesity in Ob/Ob mice. Endocrinology.

[42] Li P, Fan C, Cai Y, Fang S, Zeng Y, Zhang Y (2020). Transplantation of brown adipose tissue up-regulates miR-99a to ameliorate liver metabolic disorders in diabetic mice by targeting NOX4. Adipocyte.

[43] Liu H, Liu X, Wang L, Sheng N (2017). Brown adipose tissue transplantation ameliorates male fertility impairment caused by diet-induced obesity. Obes Res Clin Pract.

[44] Benjamin EJ, Virani SS, Callaway CW, Chamberlain AM, Chang AR, Cheng S (2018). Heart disease and stroke statistics-2018 update: a report from the american heart association. Circulation.

[45] Henareh L, Agewall S (2012). 2-h postchallenge plasma glucose predicts cardiovascular events in patients with myocardial infarction without known diabetes mellitus. Cardiovasc Diabetol.

[46] Bartnik M, Malmberg K, Norhammar A, Tenerz A, Ohrvik J, Rydén L (2004). Newly detected abnormal glucose tolerance: an important predictor of long-term outcome after myocardial infarction. Eur Heart J.

[47] Knudsen EC, Seljeflot I, Abdelnoor M, Eritsland J, Mangschau A, Müller C (2011). Impact of newly diagnosed abnormal glucose regulation on long-term prognosis in low risk patients with ST-elevation myocardial infarction: A follow-up study. BMC Endocr Disord.

[48] Del Buono MG, Arena R, Borlaug BA, Carbone S, Canada JM, Kirkman DL (2019). Exercise intolerance in patients with heart failure: JACC state-of-the-art review. J Am Coll Cardiol.

[49] Poole DC, Richardson RS, Haykowsky MJ, Hirai DM, Musch TI (2018). Exercise limitations in heart failure with reduced and preserved ejection fraction. J Appl Physiol (1985).

[50] Xu X, Wan W, Powers AS, Li J, Ji LL, Lao S (2008). Effects of exercise training on cardiac function and myocardial remodeling in post myocardial infarction rats. J Mol Cell Cardiol.

[51] Lele SS, Thomson HL, Seo H, Belenkie I, McKenna WJ, Frenneaux MP (1995). Exercise capacity in hypertrophic cardiomyopathy. Role of stroke volume limitation, heart rate, and diastolic filling characteristics. Circulation.

[52] Le VV, Perez MV, Wheeler MT, Myers J, Schnittger I, Ashley EA (2009). Mechanisms of exercise intolerance in patients with hypertrophic cardiomyopathy. Am Heart J.

[53] Gruzdeva O, Uchasova E, Dyleva Y, Belik E, Shurygina E, Barbarash O (2013). Plasminogen activator inhibitor-1, free fatty acids, and insulin resistance in patients with myocardial infarction. Diabetes Metab Syndr Obes.

[54] Vasamsetti SB, Coppin E, Zhang X, Florentin J, Koul S, Götberg M, et al. Apoptosis of hematopoietic progenitor-derived adipose tissue-resident macrophages contributes to insulin resistance after myocardial infarction. Sci Transl Med. 2020. 22;12:eaaw0638. 10.1126/scitranslmed.aaw0638 PMC7813555 32718989

[55] Wang J, Liu B, Han H, Yuan Q, Xue M, Xu F (2015). Acute hepatic insulin resistance contributes to hyperglycemia in rats following myocardial infarction. Mol Med.

[56] Cohen P, Kajimura S (2021). The cellular and functional complexity of thermogenic fat. Nat Rev Mol Cell Biol.

[57] Engin A (2017). The pathogenesis of obesity-associated adipose tissue inflammation. Adv Exp Med Biol.

[58] Tzanavari T, Giannogonas P, Karalis KP (2010). TNF-alpha and obesity. Curr Dir Autoimmun.

[59] Huang X, Liu G, Guo J, Su Z (2018). The PI3K/AKT pathway in obesity and type 2 diabetes. Int J Biol Sci.

[60] Plomgaard P, Bouzakri K, Krogh-Madsen R, Mittendorfer B, Zierath JR, Pedersen BK (2005). Tumor necrosis factor-alpha induces skeletal muscle insulin resistance in healthy human subjects via inhibition of Akt substrate 160 phosphorylation. Diabetes.

[61] Wang T, Kusudo T, Takeuchi T, Yamashita Y, Kontani Y, Okamatsu Y (2013). Evodiamine inhibits insulin-stimulated mTOR-S6K activation and IRS1 serine phosphorylation in adipocytes and improves glucose tolerance in obese/diabetic mice. PLoS One.

[62] Zhang J, Gao Z, Yin J, Quon MJ, Ye J (2008). S6K directly phosphorylates IRS-1 on Ser-270 to promote insulin resistance in response to TNF-(alpha) signaling through IKK2. J Biol Chem.

[63] Altarejos JY, Montminy M (2011). CREB and the CRTC co-activators: sensors for hormonal and metabolic signals. Nat Rev Mol Cell Biol.

[64] Herzig S, Long F, Jhala US, Hedrick S, Quinn R, Bauer A (2001). CREB regulates hepatic gluconeogenesis through the coactivator PGC-1. Nature.

[65] Qi L, Saberi M, Zmuda E, Wang Y, Altarejos J, Zhang X (2009). Adipocyte CREB promotes insulin resistance in obesity. Cell Metab.

