# Supplementary material for: Brown adipose tissue prevents glucose intolerance and cardiac remodeling in high-fat-fed mice after a mild myocardial infarction
Source: Int J Obes (Lond). 2021 Oct 29;46(2):350–8. doi: 10.1038/s41366-021-00999-9 (PMC8794788; doi:10.1038/s41366-021-00999-9)
Supplement: Supplementary file 1 — Supplemental Figure Legends [file 41366_2021_999_MOESM1_ESM.docx]

**Supplemental Figure Legends**

**Supplemental Figure 1. Baseline cardiac function is not altered among groups.**  (A) Baseline ejection fraction (2 weeks prior to MI) of Chow-fed (n=11), Sham (n=21), and +BAT (n=22) mice. (B) Ejection fraction at 22 weeks post-MI of Sham (n=2), Sham-MI (n=4) and +BAT-MI mice (n=7), which were excluded from the study because they were in heart failure (EF less than 35%). (C) % of survival at 22 weeks post-MI of Sham and +BAT-MI mice. Data are presented as mean + S.E.M. One-way ANOVA was used with Tukey’s multiple comparisons tests.

**Supplemental Figure 2. Body composition 22 weeks post MI is not affected by either MI or +BAT transplantation.** (A) Glucose tolerance and (B) insulin tolerance of Sham (n=33) and +BAT (n=33) mice prior to MI. (C) Body weight, (D) fat mass, and (E) lean Mass pre-MI and 4, 10 and 22 weeks post MI. (F) Average food intake (g/mouse/day) over the first 10 weeks post MI in Sham (n=9), Sham-MI (n=6), +BAT (n=10), and +BAT-MI (n=5). Data are presented as mean + S.E.M. T-test was used for the GTT and ITT comparison. Two-way ANOVA was used with Tukey’s multiple comparisons tests for body composition and food intake; (*p<0.05 vs. Sham).

**Supplemental Figure 3**. **Minimal effect of either MI or +BAT on gene expression in pgWAT and liver.**  Quantitative PCR (qPCR) were performed on tissue isolated from Sham, +BAT, Sham-MI, and +BAT-MI mice that were sacrificed at 52 weeks of age (22 weeks post-MI). For pgWAT, we assessed the expression of genes for (A) inflammation, (B) fibrosis, (C) glucose metabolism, (D) lipid metabolism, (E) TCA cycle, and (F) antioxidant defense. For liver, we assessed the expression of genes for (G) inflammation, (H) fibrosis, (I) glucose metabolism, (J) lipids metabolism MI of Sham (n=8), Sham-MI (n=3-5), +BAT (n=5-8), and +BAT-MI (n=4-5) mice. Data are presented as mean + S.E.M. Two-way ANOVA was used with Tukey’s multiple comparisons tests. *symbols represent difference as compared to Sham (*p<0.05). # Symbols represent difference as compared to Sham-MI (#p<0.05). % symbols represent difference as compared to +BAT-MI mice (%p<0.05).

**Supplemental Figure 4**. **Minimal effect of either MI or +BAT on gene expression in liver and heart.** Quantitative PCR (qPCR) was performed on tissue isolated from Sham, +BAT, Sham-MI, and +BAT-MI mice that were sacrificed at 52 weeks of age (22 weeks post-MI). For liver, we assessed the expression of genes for (A) TCA cycle and Mitochondrial Function, and (B) antioxidant defense. For heart, we assessed the expression of genes for (C) inflammation, (D) fibrosis, (E) insulin signaling, (F) glucose metabolism, (G) lipid metabolism, (H) mitochondrial biogenesis and TCA cycle, (I) antioxidant defense, and (J) heart function of Sham (n=8), Sham-MI (n=3-5), +BAT (n=5-8), and +BAT-MI (n=4-5) mice. Data are presented as mean + S.E.M. Two-way ANOVA was used with Tukey’s multiple comparisons tests. *Symbols represent difference as compared to Sham (*p<0.05). # Symbols represent difference as compared to Sham-MI (#p<0.05). % symbols represent difference as compared to +BAT (%p<0.05).

**Supplemental Figure 5**. **Minimal effect of either MI or +BAT on gene expression in TA and BAT.** Quantitative PCR (qPCR) were performed on tissue isolated from Sham, +BAT, Sham-MI, and +BAT-MI mice that were sacrificed at 52 weeks of age (22 weeks post-MI). For TA, we assessed the expression of genes for (A) inflammation and fibrosis (B) insulin signaling and glucose metabolism, (C) mitochondrial biogenesis, and (D) antioxidant defense. For iBAT, we assessed the expression of genes for (E) inflammation and fibrosis, (F) insulin signaling and glucose metabolism, (G) mitochondrial biogenesis and thermogenesis, and (H) antioxidant defense of Sham (n=8), Sham-MI (n=3-5), +BAT (n=5-8), and +BAT-MI (n=4-5) mice. Data are presented as mean + S.E.M. Two-way ANOVA was used with Tukey’s multiple comparisons tests. *Symbols represent difference as compared to Sham (*p<0.05). # Symbols represent difference as compared to Sham-MI (#p<0.05). % symbols represent difference as compared to +BAT (%p<0.05).
