# Supplementary material for: Brown adipose tissue prevents glucose intolerance and cardiac remodeling in high-fat-fed mice after a mild myocardial infarction
Source: Int J Obes (Lond). 2021 Oct 29;46(2):350–8. doi: 10.1038/s41366-021-00999-9 (PMC8794788; doi:10.1038/s41366-021-00999-9)
Supplement: Supplementary file 2 — Supplemental Tables [file 41366_2021_999_MOESM2_ESM.docx]

| Gene | Forward Sequence | Reverse Sequence |
| --- | --- | --- |
| Acox | GGGAGTGCTACGGGTTACATG | CCGATATCCCCAACAGTGATG |
| Adiponectin | AGATGGCACTCCTGGAGAGAAG | ACATAAGCGGCTTCTCCAGGCT |
| Akt | GGACTACTTGCACTCCGAGAAG | CATAGTGGCACCGTCCTTGATC |
| Aldoa | GCGACCACCATGTCTATCTG | GAAAGTGACCCCAGTGACAG |
| Calcineurin | CGGAAACCATGAATGTAGGCACC | GAAGGCATCCATACAGGCGTCA |
| Cd34 | AGGACAGCAGTAAGACCACACC | GTGTGGAGTTCCAGAGCCTGAA |
| Cd36 | TGGAGCTGTTATTGGTGCAG | TGGGTTTTGCACATCAAAGA |
| Cd68 | GGCGGTGGAATACAATGTGTCC | AGCAGGTCAAGGTGAACAGCTG |
| Cidea | GGTGGACACAGAGGAGTTCTTTC | CGAAGGTGACTCTGGCTATTCC |
| Citrate Synthase | GACTACATCTGGAACACACTCAATTCA | CGAGGGTCAGTCTTCCTCAGTAC |
| Col I | CCTCAGGGTATTGCTGGACAAC | CAGAAGGACCTTGTTTGCCAGG |
| Col II | TTCTGTGGGTCCTGCTGGGAAA | TTGTCACCTCGGATGCCTTGAG |
| Cpt1a | AAAGATCAATCGGACCCTAGACA | CAGCGAGTAGCGCATAGTCA |
| Creb | CACAGACCACTGATGGACAGCA | AGGACGCCATAACAACTCCAGG |
| Csf1 | GCCTCCTGTTCTACAGGAAG | ACTGGCAGTTCCTGTCTGT |
| Csf1R | TGGATGCCTGTGAATTCTG | GTGGGTGTCATTAACCTGC |
| Cypd | AGATGTCAAATTGGCAGGGGG | TGCGCTTTTCGGTATAGTGCT |
| Ddr2 | TCATCCTGTGGAGGCAGTTCTG | CTGTTCACTTGGTGATGAGGAGC |
| Elovl3 | ACTTCGAGACGTTTCAGGACTT | CCACTATGAGAAATGAGCTTACCC |
| Elovl6 | CGGCATCTGATGAACAAGCGAG | GTACAGCATGTAAGCACCAGTTC |
| Eno3 | CTGTGCCTGCCTTTAATGTG | CTTCCCATACTTGGCCTTGA |
| Ephx2 | CAATGGTTCCTGTCGTCCAGTAG | AGTTCTCCACCTGGACCAAGTC |
| F4/80 | CGTGTTGTTGGTGGCACTGTGA | CCACATCAGTGTTCCAGGAGAC |
| Fabp3 | AGAGTTCGACGAGGTGACAGCA | TTGTCTCCTGCCCGTTCCACTT |
| Fabp5 | GACGACTGTGTTCTCTTGTAACC | TGTTATCGTGCTCTCCTTCCCG |
| Fatp1 | TGCCACAGATCGGCGAGTTCTA | AGTGGCTCCATCGTGTCCTCAT |
| Fatp4 | GACTTCTCCAGCCGTTTCCACA | CAAAGGACAGGATGCGGCTATTG |
| Fox01 | CTACGAGTGGATGGTGAAGAGC | CCAGTTCCTTCATTCTGCACTCG |
| Gdf15 | AGCCGAGAGGACTCGAACTCAG | GGTTGACGCGGAGTAGCAGCT |
| Glut2 | GTTGGAAGAGGAAGTCAGGGCA | ATCACGGAGACCTTCTGCTCAG |
| Glut4 | ATCATCCGGAACCTGGAGG | CGGTCAGGCGCTTTAGACTC |
| Gpi1 | ATGGGCATATTCTGGTGGAC | CCCGATTCTCGGTGTAGTTG |
| Gpx1 | AGTTCCAGGCAATGTCGTTGCG | CGCTCTTTACCTTCCTGCGGAA |
| Gpx4 | CTTATCCAGGCAGACCATGTGC | CCTCTGCTGCAAGAGCCTCCC |
| Gyk | CAAATGCAAGCAGGACGATG | GGCCCCAGCTTTCATTAGG |
| HkII | AGAGAACAAGGGCGAGGAG | GGAAGCGGACATCACAATC |
| Hsl | GCTCATCTCCTATGACCTACGG | TCCGTGGATGTGAACAACCAGG |
| Icam | AAACCAGACCCTGGAACTGCAC | GCCTGGCATTTCAGAGTCTGCT |
| Idh3a | GCAGGACTGATTGGAGGTCTTG | GCCATGTCCTTGCCTGCAATGT |
| Il10 | CGGGAAGACAATAACTGCACCC | CGGTTAGCAGTATGTTGTCCAGC |
| Il1β | TGGACCTTCCAGGATGAGGACA | GTTCATCTCGGAGCCTGTAGTG |
| Il6 | TACCACTTCACAAGTCGGAGGC | CTGCAAGTGCATCATCGTTGTTC |
| Irs1 | TGTCACCCAGTGGTAGTTGCTC | CTCTCAACAGGAGGTTTGGCATG |
| Leptin Receptor | CTTTCCTGTGGACAGAACCAGC | AGCACTGAGTGACTCCACAGCA |
| Lpl | GATGCCCTACAAAGTGTTCCA | AAATCTCGAAGGCCTGGTTG |
| Mapk8 | CGCCTTATGTGGTGACTCGCTA | TCCTGGAAAGAGGATTTTGTGGC |
| Mcp1 | GCTACAAGAGGATCACCAGCAG | GTCTGGACCCATTCCTTCTTGG |
| Mdh2 | TCACTCCTGCTGAAGAACAGCC | CCTTTGAGGCAATCTGGCAACTG |
| Mfn1 | GCAGACAGCACATGGAGAGA | GATCCGATTCCGAGCTTCCG |
| Mfn2 | TGCACCGCCATATAGAGGAAG | TCTGCAGTGAACTGGAATG |
| Mmp2 | GAGACCATGCAGTCAGCTCTAG | TAGAGCTGCCTCTTGTCTGGTC |
| Mmp9 | GCTGACTACGATAAGGACGGCA | TAGTGGTGCAGGCAGAGTAGGA |
| Mtor | AGAAGGGTCTCCAAGGACGACT | GCAGGACACAAAGGCAGCATTG |
| Nfat | ACTTCACAGCGGAGTCCAAGGT | GGATGTGCTTGTTCCGATACTCG |
| NfkB | GCTGCCAAAGAAGGACACGACA | GGCAGGCTATTGCTCATCACAG |
| Nrf1 | CAACAGGGAAGAAACGGAAA | GCACCACATTCTCCAAAGGT |
| Nrf2 | AGGTTGCCCACATTCCCAAACAAG | TTGCTCCATGTCCTGCTCTATGCT |
| Ogdha | GGTGTCGTCAATCAGCCTGAGT | ATCCAGCCAGTGCTTGATGTGC |
| Opa1 | ACCTTGCCAGTTTAGCTCCC | TTGGGACCTGCAGTGAAGAA |
| Pcx | GGATGACCTCACAGCCAAGCAT | GCAATCGAAGGCTGCGTACAGT |
| Pdh1a | GTGAGAACAACCGCTATGGCATG | CGCAAACTTTGTTGCCTCTCGG |
| Pfk1 | CCATCAGCAACAATGTGCCTGG | TGAGGCTGACTGCTTGATGCGA |
| Pfkm | CTGGTGCTGAGGAATGAGAA | TTCCTGTCAAAGGGAGTTGG |
| Pfkp | AAGCTATCGGTGTCCTGACC | TCCCACCCACTTGCAGAAT |
| Pgam1 | GACGATCTTATGATGTCCCACC | GTACCTGCGATCCTTGCTGA |
| Pgc1α | GAATCAAGCCACTACAGACACCG | CATCCCTCTTGAGCCTTTCGTG |
| Pgk1 | GAGCCTCACTGTCCAAACTA | CTTTAGCGCCTCCCAAGATA |
| Pgs1 | CTGGACTTCACCAGAGGCTCAA | CCGATGGTCTCATTGAAGCGCT |
| Pi3k | GATCGCTTGCTGTGAGCTGGAT | GGATGTTTCCCAATGCCCTGGA |
| Pnpla3 | AGACAAGGTGCCAGTCAGCCTA | GAGGTTGCAGACTTTGCTCAGG |
| Pnpla8 | CTCCAGACTCTGAGGAAGTTGG | CGGCATGTGAAACAATCCCAGC |
| Pparα | ACCACTACGGAGTTCACGCATG | GAATCTTGCAGCTCCGATCACAC |
| Pparγ | GTACTGTCGGTTTCAGAAGTGCC | ATCTCCGCCAACAGCTTCTCCT |
| Prdm16 | ATCCACAGCACGGGTGAAGCCAT | ACATCTGCCCACAGTCCTTGCA |
| Prdx1 | AGCAATGGTGCGCTTGGGATCT | TGCCAAGTGATTGGCGCTTCTG |
| Prdx2 | CCTTGCTGTCATCCACATTGGG | TACCTGTGGGACGCTCTGTAGA |
| S6k | AGGTGGAACCTCCCTTTAAGCC | CCAGAAAGACCTGGTTGGCACT |
| Sirt3 | GCTACATGCACGGTCTGTCGAA | CAATGTCGGGTTTCACAACGCC |
| Sod1 | ATGAGGTCCTGCACTGGTACAG | GGTGAACCAGTTGTGTTGTCAGG |
| Sod2 | TAACGCGCAGATCATGCAGCTG | AGGCTGAAGAGCGACCTGAGTT |
| Sod3 | TGGCTGATGGTTGTACCCTGCA | GACCTGGTTGAGAAGATAGGCG |
| Srebp1 | GAACAGACACTGGCCGAGAT | GAGGCCAGAGAAGCAGAAGAG |
| Taz | CCTTATCACCGTCTCCAACCAC | CCTTGGTGAAGCAGATGTCTGC |
| Tfam | GTCCATAGGCACCGTATTGC | CCCATGCTGGAAAAACACTT |
| Tgfβ | TGATACGCCTGAGTGGCTGTCT | CACAAGAGCAGTGAGCGCTGAA |
| Timp1 | TCTTGGTTCCCTGGCGTACTCT | GTGAGTGTCACTCTCCAGTTTGC |
| Tnf-α | GGTGCCTATGTCTCAGCCTCTT | GCCATAGAACTGATGAGAGGGAG |
| Tpi | TATGGAGGTTCTGTGACTGGA | CGGTGGGAGCAGTTACTAAA |
| Ucp1 | AGGCTTCCAGTACCATTAGGT | CTGAGTGAGGCAAAGCTGATTT |
| Vcam | GCTATGAGGATGGAAGACTCTGG | ACTTGTGCAGCCACCTGAGATC |
| Vegfa | CTGCTGTAACGATGAAGCCCTG | GCTGTAGGAAGCTCATCTCTCC |
| Vim | CGGAAAGTGGAATCCTTGCAGG | AGCAGTGAGGTCAGGCTTGGAA |

**Supplemental Table 1:** qPCR forward and reverse primer sequences.

| **Parameter** | **Chow-fed** | **Sham** | **+BAT** |
| --- | --- | --- | --- |
| Ejection Fraction (%) | 54.44 ± 1.5 | 55.6 ± 1.6 | 53.6 ± 0.9 |
| Fraction Shortening (%) | 25.8 ± 1 | 27.7 ± 1.3 | 28.7 ± 0.6 |
| Stroke Volume (μL) | 30.7 ± 1.2 | 42.5 ± 2.4^$^ | 45.5 ± 1.2^$^ |
| Cardiac Output (mL/min) | 13.6 ± 0.6 | 19.8 ± 1.3^$^ | 19.9 ± 0.8^$^ |
| LVM (mg) | 106.6 ± 4.7 | 127.9 ± 4.7^$^ | 131.8 ± 1.4^$^ |
| LVM;c (mg) | 85.3 ± 3.8 | 105.6 ± 4.8^$^ | 107.2 ± 4.3^$^ |
| Diameter;s (mm) | 2.78 ± 0.08 | 3.04 ± 0.08^$^ | 3.03 ± 0.05^$^ |
| Diameter;d (mm) | 3.75 ± 0.07 | 4.2 ± 0.08^$^ | 4.3 ± 0.05^$^ |
| Volume;s (μL) | 29.6 ± 2.1 | 36.9 ± 2.2^$^ | 36.3 ± 1.4 |
| Volume;d (μL) | 60.3 ± 2.7 | 80 ± 3.2^$^ | 81.6 ± 2.2^$^ |
| LVAW;s (mm) | 1.22 ± 0.04 | 1.27 ± 0.02 | 1.28 ± 0.03 |
| LVAW;d (mm) | 0.84 ± 0.02 | 0.79 ± 0.04 | 0.81 ± 0.04 |
| LVPW;s (mm) | 1.07 ± 0.05 | 1.06 ± 0.05 | 1.11 ± 0.03 |
| LVPW;d (mm) | 0.77 ± 0.04 | 0.81 ± 0.03 | 0.8 ± 0.03 |

**Supplemental Table 2.** Baseline echocardiography parameters. Baseline cardiac parameters of mice that were EF over 35% at the end of the protocol (22 weeks post-MI) measured by echocardiography at 2 weeks before MI: Ejection fraction, fraction shortening, stroke volume, cardiac output, left ventricle mass (LVM), left ventricle mass corrected (LVM;c), end systolic diameter (Diameter;s), end diastolic diameter (Diameter;d), end systolic volume (Volume;s), end diastolic volume (Volume;d), left ventricle anterior wall systolic (LVAW;s), left ventricle anterior wall diastolic (LVAW;d), left ventricle posterior wall systolic (LVPW;s), and left ventricle posterior wall diastolic (LVPW;d) in Chow-fed (n=14), Sham (n=15) and +BAT(n=13). One-way ANOVA was used with Tukey’s multiple comparisons tests. $ Symbols represent difference vs. Chow-fed mice.

| **Parameter** | **Chow-fed** | **Sham** | **Sham-MI** | **+BAT** | **+BAT-MI** |
| --- | --- | --- | --- | --- | --- |
| Ejection Fraction (%) | 51.5 ± 2.04 | 45.7 ± 1.67 | 48.8 ± 2.99 | 48.3 ± 1.96 | 46.1 ± 2.94 |
| Fraction Shortening (%) | 26.3 ± 1.27 | 22.6 ± 0.99 | 24.7 ± 1.78 | 24.2 ± 1.19 | 23 ± 1.65 |
| Stroke Volume (μL) | 43.3 ± 1.26 | 39.8 ± 3.39 | 45.6 ± 3.3 | 41.8 ± 3.07 | 45.2 ± 2.78 |
| Cardiac Output (mL/min) | 17.8 ± 0.72 | 18.7 ± 1.62 | 23.5 ± 1.68^$^ | 17.3 ± 1.22 | 22.2 ± 0.55 |
| LVM (mg) | 140 ± 5.4 | 145.4 ± 6.4 | 172.8 ± 9.4^$^ | 138.3 ± 5.3 | 152 ± 17.3 |
| LVM;c (mg) | 112 ± 4.3 | 116.4 ± 5.1 | 138.2 ± 7.5^$^ | 110.6 ± 4.2 | 121.6 ± 13.9 |
| Diameter;s (mm) | 3.21 ± 0.1 | 3.37 ± 0.09 | 3.41 ± 0.17 | 3.29 ± 0.07 | 3.59 ± 0.26 |
| Diameter;d (mm) | 4.34 ± 0.08 | 4.36 ± 0.13 | 4.52 ± 0.15 | 4.35 ± 0.09 | 4.64 ± 0.24 |
| Volume;s (μL) | 42.2 ± 3.02 | 47.1 ± 3.09 | 48.8 ± 5.82 | 44.2 ± 2.19 | 56 ± 9.63 |
| Volume;d (μL) | 85.5 ± 3.38 | 86.9 ± 5.75 | 94.5 ± 7.47 | 86 ± 4.31 | 101.2 ± 12.4 |
| LVAW;s (mm) | 1.38 ± 0.04 | 1.21 ± 0.04 | 1.41 ± 0.09 | 1.33 ± 0.05 | 1.21 ± 0.08 |
| LVAW;d (mm) | 0.91 ± 0.03 | 0.85 ± 0.03 | 1.05 ± 0.08 | 0.88 ± 0.06 | 0.84 ± 0.03 |
| LVPW;s (mm) | 1.06 ± 0.03 | 1.04 ± 0.05 | 1.03 ± 0.07 | 0.93 ± 0.07 | 0.92 ± 0.05 |
| LVPW;d (mm) | 0.73 ± 0.03 | 0.83 ± 0.04 | 0.78 ± 0.02 | 0.75 ± 0.01 | 0.75 ± 0.03 |

**Supplemental Table 3.** Echocardiography parameters 22 weeks post-MI. Cardiac parameters of mice with EF over 35% measured by echocardiography at 22 weeks post MI: Ejection fraction, fraction shortening, stroke volume, cardiac output, left ventricle mass (LVM), left ventricle mass corrected (LVM;c), end systolic diameter (Diameter;s), end diastolic diameter (Diameter;d), end systolic volume (Volume;s), end diastolic volume (Volume;d), left ventricle anterior wall systolic (LVAW;s), left ventricle anterior wall diastolic (LVAW;d), left ventricle posterior wall systolic (LVPW;s), and left ventricle posterior wall diastolic (LVPW;d) in Chow-fed (n=14), Sham (n=9), Sham-MI (n=6), +BAT(n=9), and +BAT-MI (n=5). One-way ANOVA was used with Tukey’s multiple comparisons tests. $ Symbols represent difference vs. Chow-fed mice.
