# Supplementary figures and images for: Brown adipose tissue prevents glucose intolerance and cardiac remodeling in high-fat-fed mice after a mild myocardial infarction
Source: Int J Obes (Lond). 2021 Oct 29;46(2):350–8. doi: 10.1038/s41366-021-00999-9 (PMC8794788; doi:10.1038/s41366-021-00999-9)

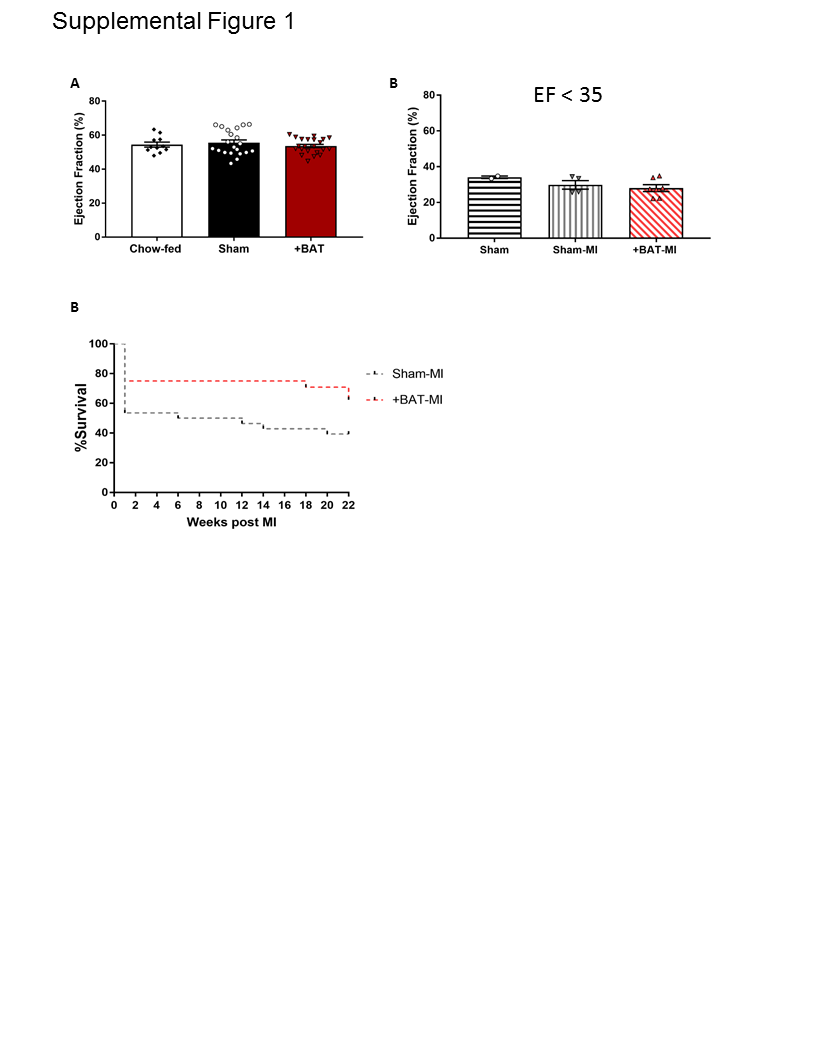

Supplement: Supplementary file 3 — Supplemental Figure 1 [file 41366_2021_999_MOESM3_ESM.tif]

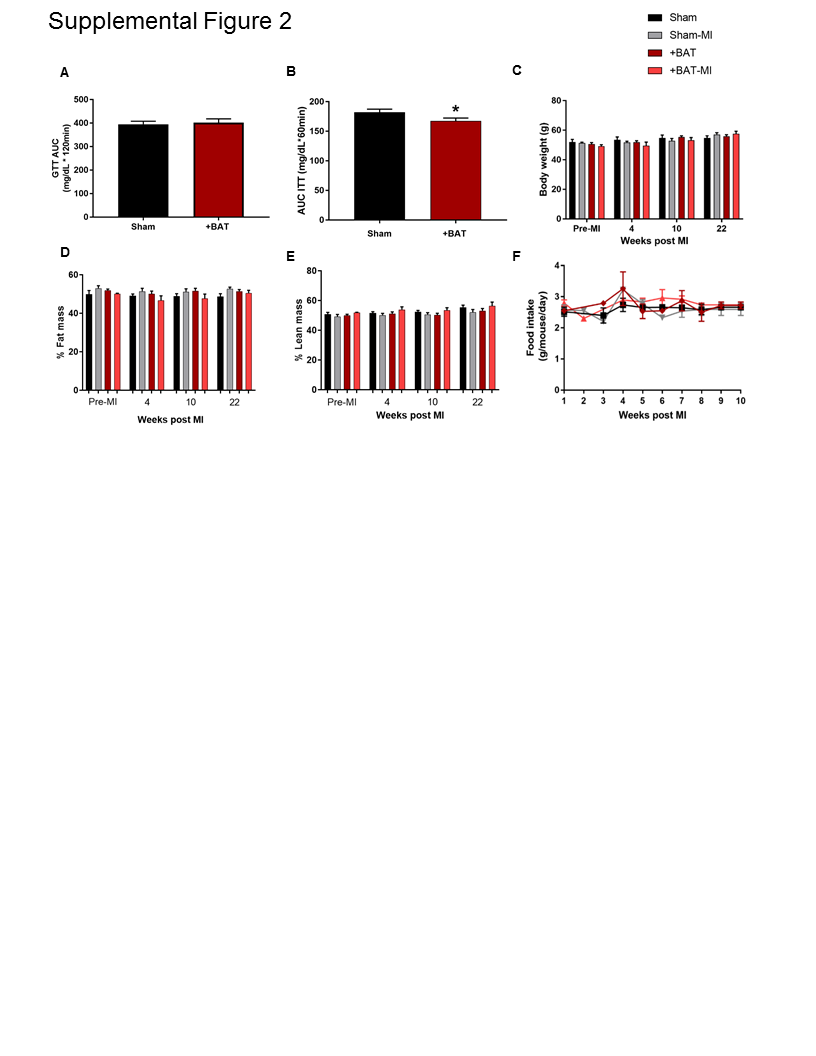

Supplement: Supplementary file 4 — Supplemental Figure 2 [file 41366_2021_999_MOESM4_ESM.tif]

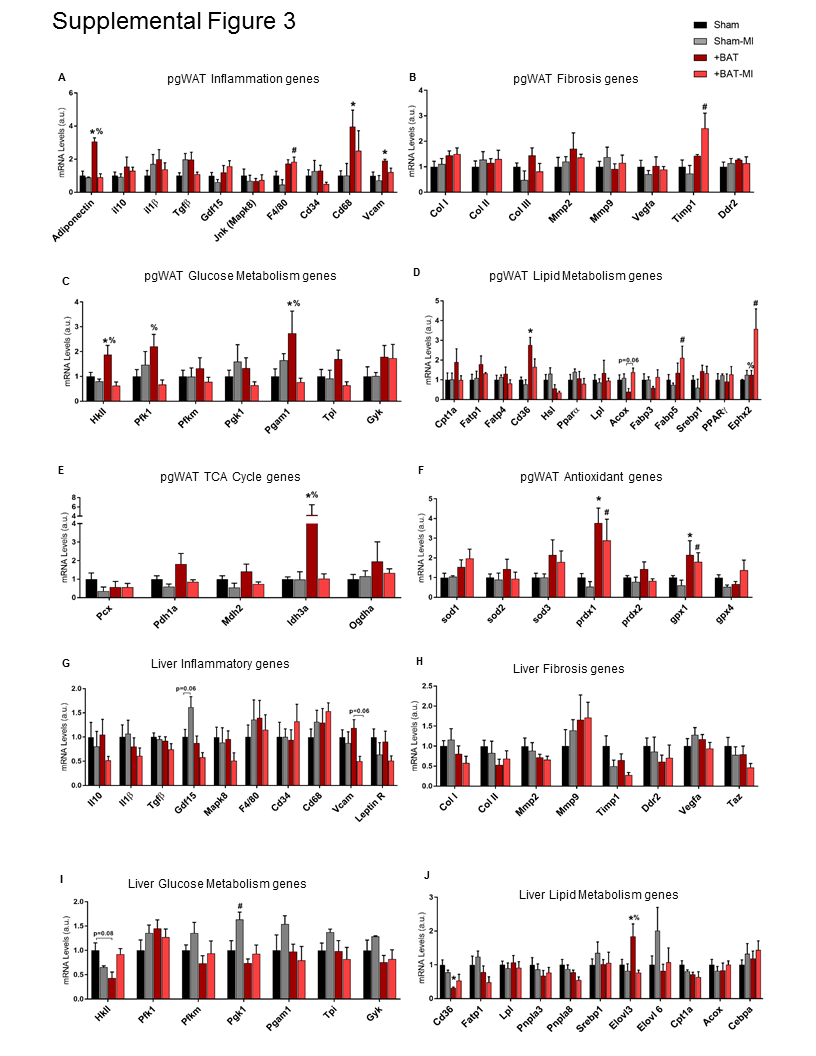

Supplement: Supplementary file 5 — Supplemental Figure 3 [file 41366_2021_999_MOESM5_ESM.tif]

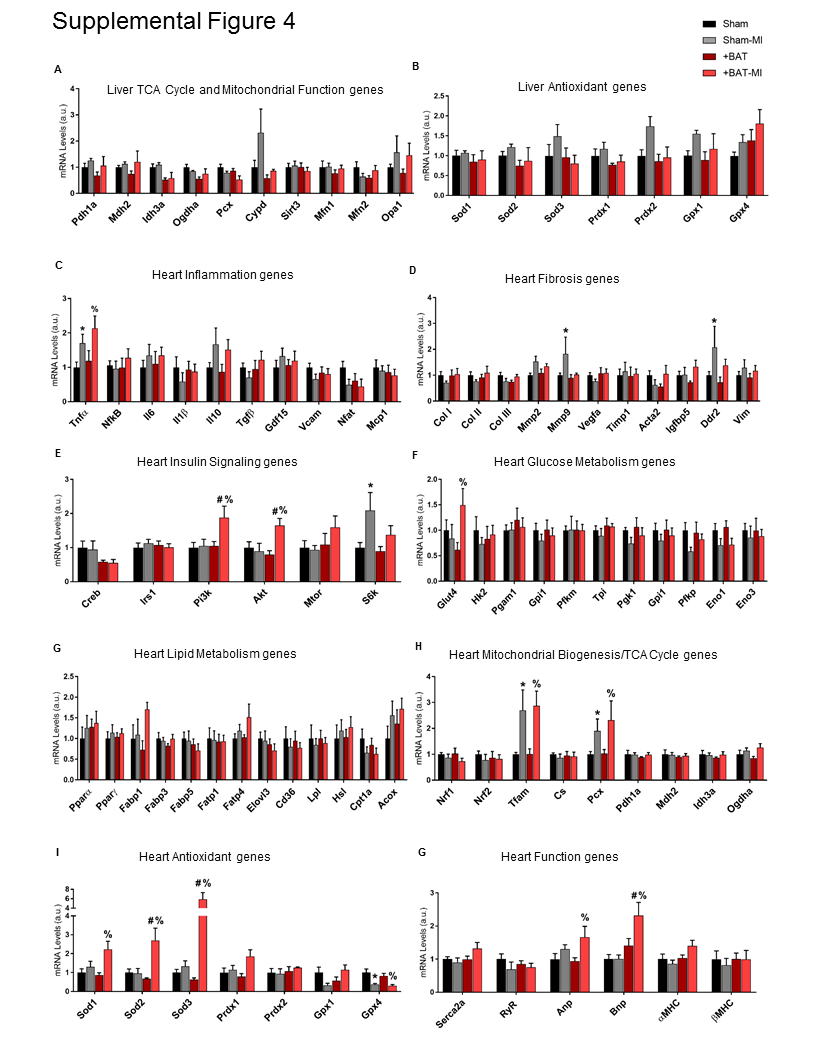

Supplement: Supplementary file 6 — Supplemental Figure 4 [file 41366_2021_999_MOESM6_ESM.tif]

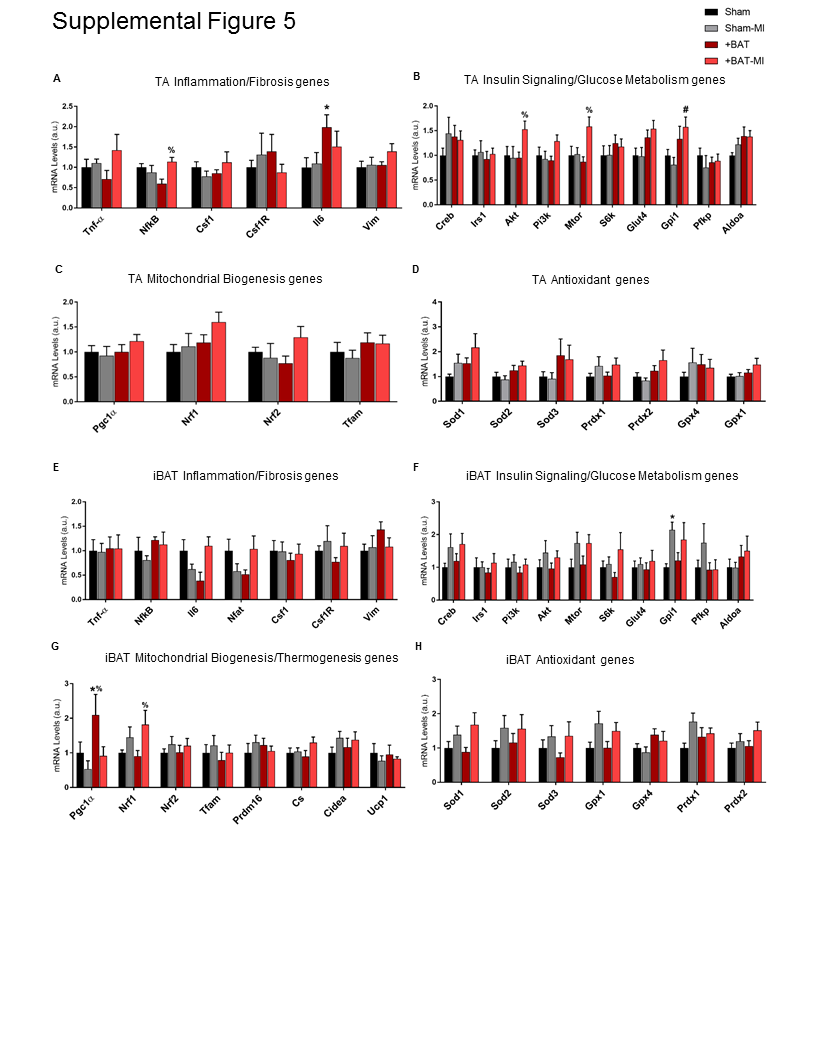

Supplement: Supplementary file 7 — Supplemental Figure 5 [file 41366_2021_999_MOESM7_ESM.tif]
